# Supplementary material for: Enhanced Fluctuations in Facial Pore Size, Redness, and TEWL Caused by Mask Usage Are Normalized by the Application of a Moisturizer
Source: J Clin Med. 2022 Apr 11;11(8):2121. doi: 10.3390/jcm11082121 (PMC9030563; doi:10.3390/jcm11082121)

Supplementary Figure S1

Morning-to-evening fluctuation of skin hydration, TEWL, pore size, and redness. The pore size was significantly larger in the afternoon when the subjects wore their masks. A significant increase in TEWL was seen in the evening.

***: P < 0.05**

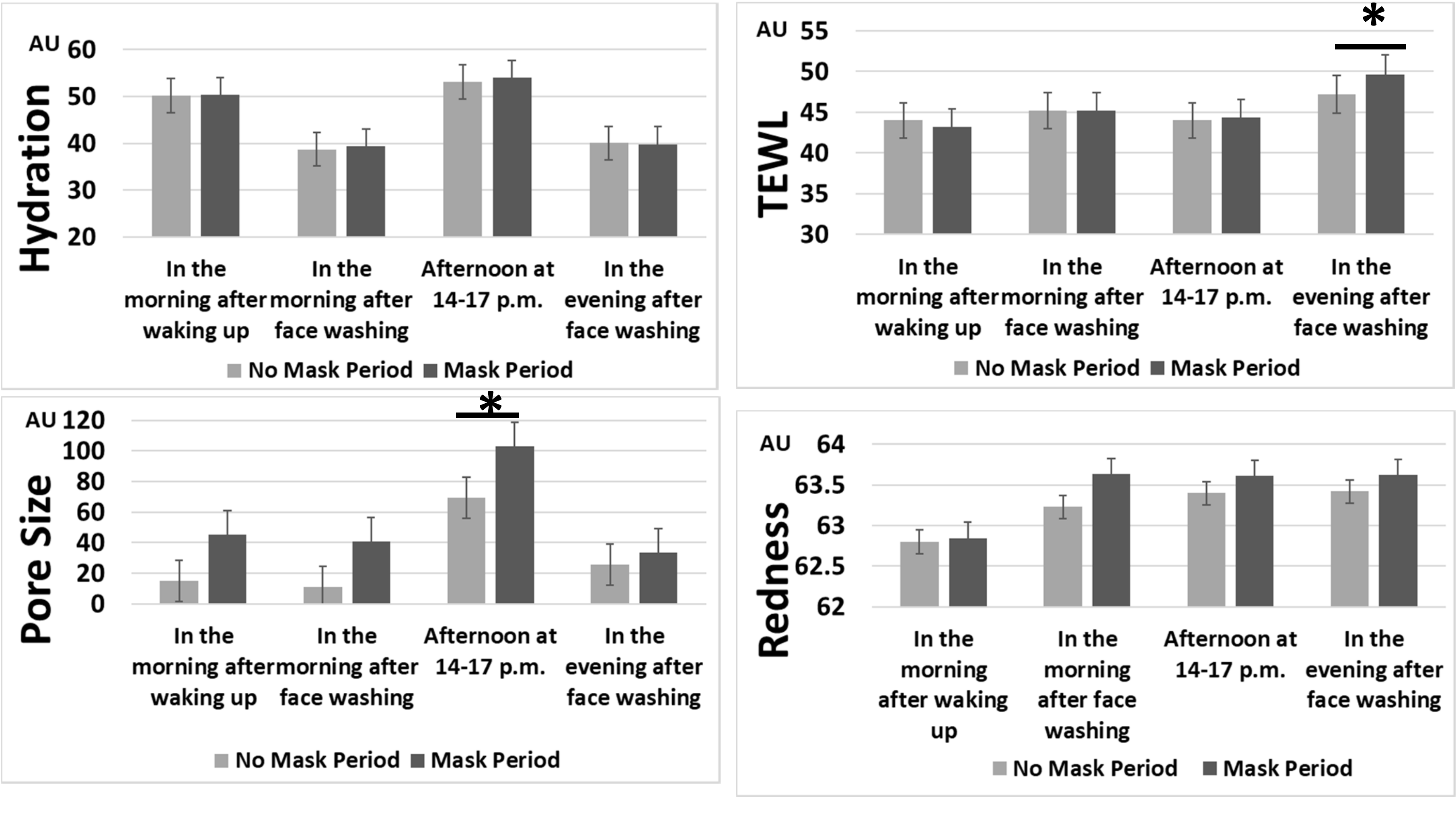

Supplementary Figure S2

Skin parameters showed morning-to-evening fluctuation.
The intra-day Δ fluctuation was calculated as |X2-X1| + |X2-X3| + |X4-X3|.

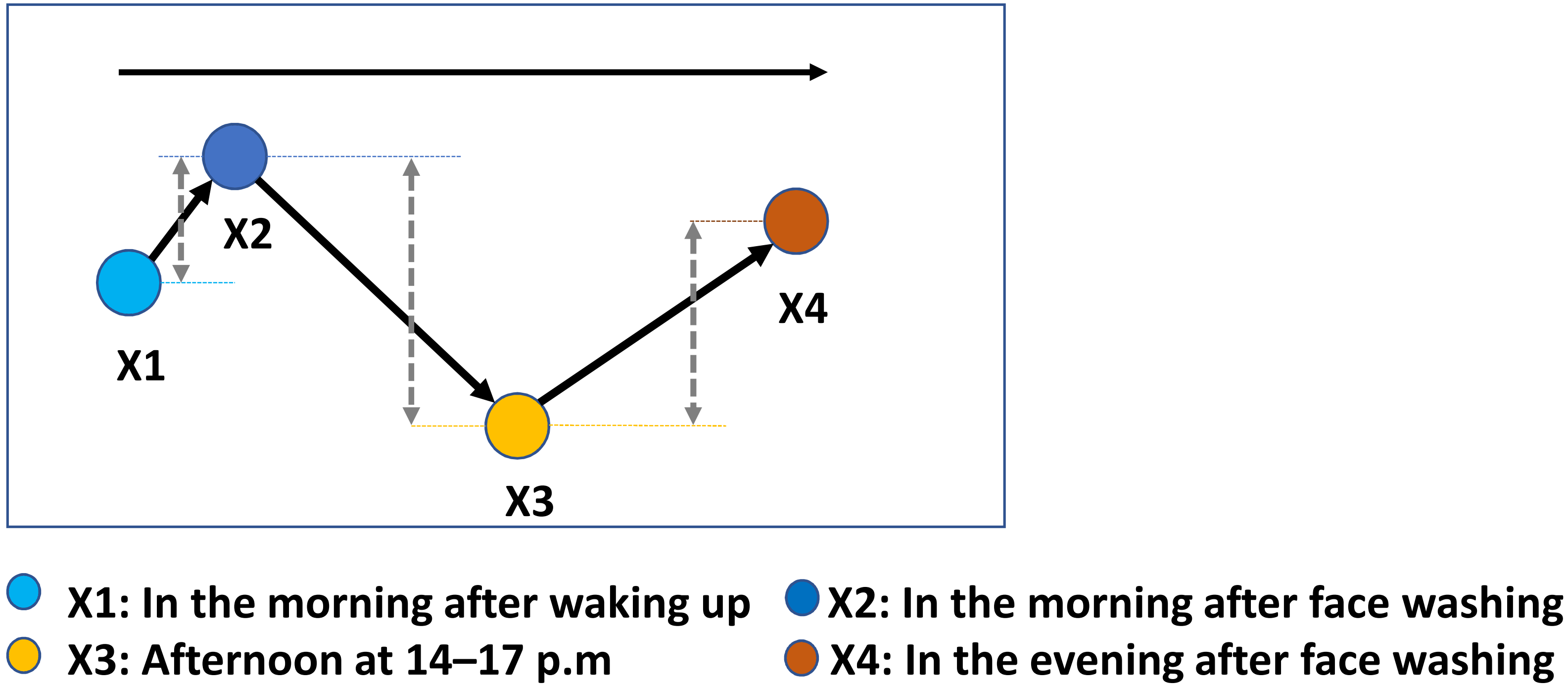

Supplementary Figure S3

The intra-day Δ fluctuation of pore size was significantly correlated with the intra-day Δ fluctuation of redness.

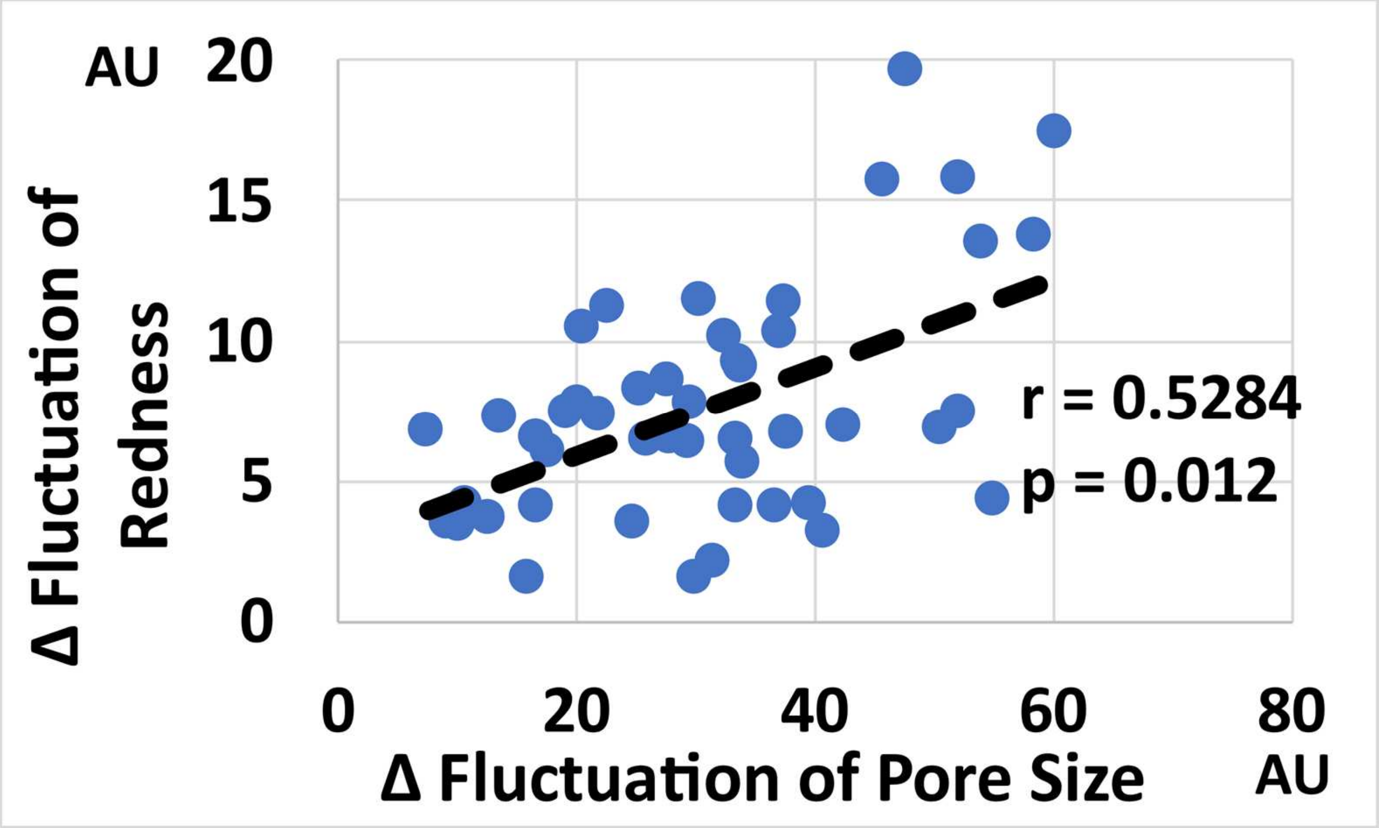

Supplementary Figure S4

The intra-day averages of skin hydration and TEWL were significantly improved by treatment with a GFF-containing moisturizer.

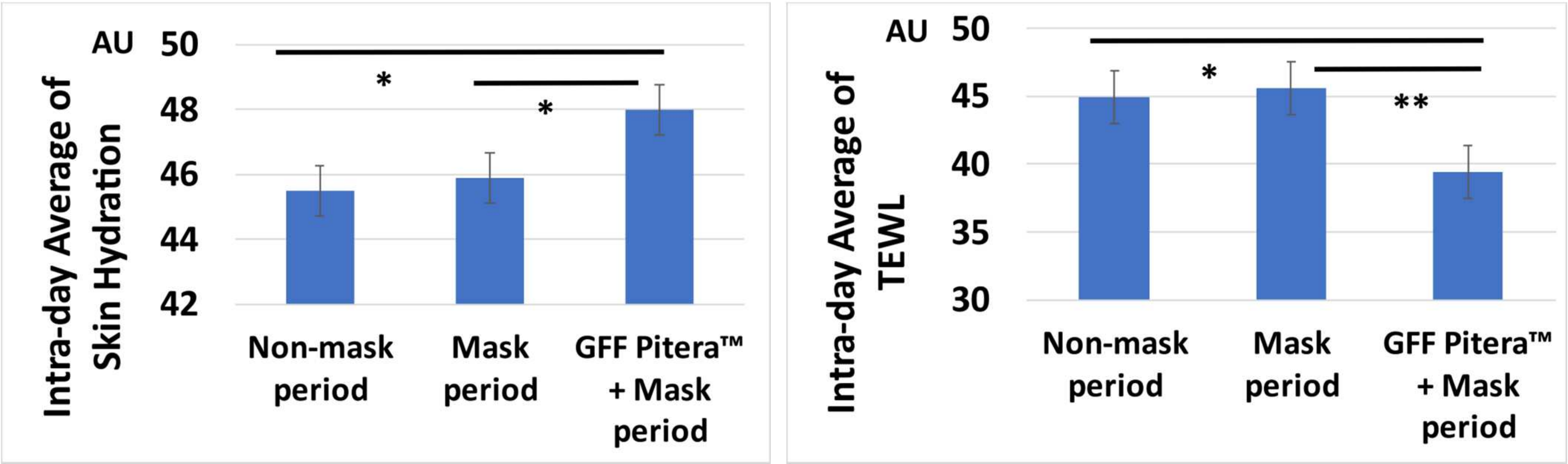

Supplement: Supplementary file 1 [file jcm-11-02121-s001.zip › jcm-1652690-SI.pdf]
